# Moxifloxacin Prophylaxis against MDR TB, New York, New York, USA 

**DOI:** 10.3201/eid2103.141313

**Published:** 2015-03

**Authors:** Lisa Trieu, Douglas C. Proops, Shama D. Ahuja

**Affiliations:** New York City Department of Health and Mental Hygiene, Bureau of Tuberculosis Control, New York, New York, USA

**Keywords:** tuberculosis and other mycobacteria, fluoroquinolones, antibiotic, antimicrobials, moxifloxacin, contact tracing, latent tuberculosis, prophylaxis, outbreak, multidrug-resistant tuberculosis, MDR TB, human immunodeficiency virus, HIV, New York, bacteria

## Abstract

Contacts of persons infected with multidrug-resistant tuberculosis (MDR TB) have few prophylaxis options. Of 50 contacts of HIV- and MDR TB–positive persons who were treated with moxifloxacin, 30 completed treatment and 3 discontinued treatment because of gastrointestinal symptoms. Moxifloxacin was generally well-tolerated; further research of its efficacy against MDR TB is needed.

Limited data exist on safety of prophylaxis for contacts to persons with multidrug-resistant tuberculosis (MDR TB). All MDR TB strains are resistant to at least isoniazid and rifampin, precluding the use of these drugs for MDR TB prophylaxis. Current local, national, and international guidelines suggest using antibiotics to which the strain from the index case-patient is susceptible (*1*–*6*; Table 1); however, no randomized controlled trial has been conducted to support this recommendation. Global spread of MDR TB necessitates identification of treatment options with acceptable safety and tolerability for persons infected with drug-resistant strains. Choosing appropriate treatment for HIV-positive persons exposed to TB is even more crucial considering the increased risk among these persons for progression from TB infection to active disease (*7*–*9*).

**Table 1:** Current recommendations of international public health agencies for managing multidrug-resistant tuberculosis infection among contacts of infected persons

Recommendation
Treatment with isoniazid alone if high likelihood that the contact had prior exposure to and infection caused by a drug-susceptible case (*2**,**4*)
Treatment with >2 antimicrobial drugs to which the index case is susceptible, including pyrazinamide and ethambutol (*1**,**2**,**4**,**5*)
Treatment with pyrazinamide or ethambutol and a fluoroquinolone (*1**,**2**,**4*)
Clinical monitoring for 2 years for signs or symptoms of active disease (*2**,**3*)

In 2005, two TB outbreaks occurred in New York City (NYC) among HIV-positive persons with 2 distinct MDR TB strains. In both outbreaks, contacts were defined as 1) residents of a building on the floor on which a case-patient resided or visited during the infectious period and 2) health care staff members who provided direct care to case-patients. Eligible contacts were treated with moxifloxacin to prevent progression from TB infection to disease. We present the 9-year follow-up from these exposures and the outcomes of the treated contacts.

## The Investigation

The first outbreak we investigated occurred in a facility that provided housing and harm-reduction services to a predominantly HIV-positive, homeless, and drug-using population (site A). The first TB case-patient identified was a 53-year-old HIV-positive man residing there, in whom pulmonary TB was diagnosed by a positive (4+) acid-fast bacilli (AFB) sputum smear and positive culture. His chest radiograph showed extensive bilateral infiltrates and a large pulmonary cavity; he died 4 days after initiating treatment. Subsequent drug-susceptibility results indicated the strain was resistant to isoniazid, rifampin, ethambutol, pyrazinamide, streptomycin, rifabutin, and kanamycin. Within 3 months, TB was diagnosed in 2 additional HIV-positive residents of site A; genotype and drug-resistance phenotype matched those of the index case-patient. A contact investigation and active case finding were initiated at site A, and 3 additional MDR TB cases with matching genotype were identified.

Of 105 close contacts identified, 84 (80%) were HIV-positive, 16 (15%) were HIV-negative, and 5 (5%) had unknown HIV status (Table 2). Among the 21 contacts not known to be HIV-positive, 1 person had a positive tuberculin skin test (TST) result, had normal chest radiograph results, and started moxifloxacin prophylaxis; however, the patient was lost to follow-up after 2 months. Among the 84 HIV-positive contacts, TST results of 2 were positive and that of 1 other contact was positive after a negative result documented 3 years before. Fifty-one (61%) HIV-positive contacts were lost to follow-up or refused evaluation or prophylaxis. Before being tested, 1 (1%) contact died as a result of HIV-related causes. Of the remaining 32 (38%) HIV-positive persons, 26 (81%) started moxifloxacin prophylaxis; 16 (62%) completed treatment, 5 (19%) were lost to follow-up within 2 months (including the 3 who tested TS positive), 3 (12%) were discharged from treatment because of adverse reactions, and 2 (8%) were either medically discharged for unknown reasons or refused to continue treatment.

**Table 2:** Multidrug resistant tuberculosis contact investigation results at 2 sites, by HIV status and country of birth, New York, New York, USA*

Site	US-born, no. (%)	Foreign-born, no. (%)	Unknown country of birth, no. (%)		Total, no. (%)
Site A					
No. contacts	87	6	12		105
HIV-positive	68 (78)	4 (67)	12 (100)		84 (80)
Eligible for testing†	56 (82)	1 (25)	12 (100)		69 (82)
Tested	22 (39)	1 (100)	2 (17)		25 (36)
TST positive	3 (14)	0 (0)	0 (0)		3 (12)
Initiated treatment	24 (35)	3 (75)	2 (17)		29 (35)
Treated with moxifloxacin	21 (88)	3 (100)	2 (100)		26 (90)
Completed treatment	13 (62)	2 (67)	1 (50)		16 (62)
Treated with alternate regimen	3 (13)	0 (0)	0		3 (10)
Did not initiate treatment	44 (65)	1 (25)	10 (83)		55 (65)
Lost to follow-up	28 (64)	1 (100)	10 (100)		39 (71)
Refused evaluation or treatment	12 (27)	0 (0)	0		12 (22)
Died before testing or treatment	1 (2)	0 (0)	0		1 (2)
Physician decision to not treat	3 (7)	0 (0)	0		3 (5)
HIV-negative	14 (16)	2 (33)	0		16 (15)
Eligible for testing*	11 (79)	1 (50)	0		12 (75)
Tested	3 (27)	1 (100)	0		4 (33)
TST positive	0 (0)	1 (100)	0		1 (25)
HIV status unknown	5 (6)	0 (0)	0		5 (5)
Eligible for testing*	3 (60)	0	0		3 60)
Tested	0	0	0		0
TST positive	0	0	0		0
Site B					
No. contacts	47	1		88	136
HIV-positive	47 (100)	1 (100)		35 (40)	83 (61)
Eligible for testing*	45 (96)	0		31 (89)	76 (92)
Tested	20 (44)	NA		20 (65)	40 (53)
TST-positive	3 (15)	0		0 (0)	3 (8)
Initiated treatment	16 (34)	1 (100)		14 (40)	31(37)
Treated with pyrazinamide/moxifloxacin	12 (75)	1 (100)		11 (79)	24 (77)
Completed treatment	9 (75)	1 (100)		4 (36)	14 (58)
Treated with alternate regimen	4 (25)	0		3 (21)	7 (23)
Did not initiate treatment	31 (66)	0		21 (60)	52 (63)
Lost to follow-up	15 (48)	NA		11 (52)	26 (50)
Refused evaluation or treatment	2 (6)	NA		1 (5)	3 (6)
Died before testing or treatment	12 (39)	NA		2 (10)	14 (27)
Physician decision to not treat	2 (6)	NA		7 (33)	9 (17)
HIV status unknown	0	0		53 (60)	53 (39)
Eligible for testing*	NA	NA		28 (53)	28 (53)
Tested	NA	NA		25 (89)	25 (89)
TST positive	NA	NA		0 (0)	0

*Site A was a facility that provided housing and harm-reduction services to a predominantly HIV-positive, homeless, and drug-using population. Site B was a long-term care facility housing HIV-positive, homeless persons. TST, tuberculin skin test. †Any person who had a history of positive TST result was considered ineligible for testing.

The second outbreak occurred at a long-term care facility housing HIV-positive, previously homeless persons (site B). The index case-patient was a 49-year-old HIV-positive man for whom smear-positive (2+), culture-positive pulmonary TB was diagnosed. A TB strain resistant to isoniazid, rifampin, and rifabutin was identified; the patient died 1 month later. Contact investigation and active case finding were initiated at site B. Within 6 months of the index case-patient’s diagnosis, 5 additional TB cases were identified in 4 HIV-positive residents and 1 HIV-negative staff member. On the basis of genotype, the strain the index case-patient was diagnosed with matched the strain of the 3 residents and staff member. All isolates also had the same drug resistance phenotype. The other HIV-positive resident had a clinical diagnosis of TB meningitis (no culture results available). 

In the site B outbreak, 136 close contacts were identified (Table 2): 83 (61%) HIV-positive residents and 53 (39%) staff members with unknown HIV status. Of the 53 staff members, 22 (42%) were previously TST-positive but had normal chest radiograph results during this evaluation; 25 (47%) tested negative, and 6 (11%) were not evaluated. No staff members were eligible for prophylaxis. Of the 83 HIV-positive residents, 3 (4%) had positive TST results; 2 had documented negative results within the year before their positive result, strengthening evidence of TB transmission in site B.

Considering the drug susceptibility pattern of this strain, a combination of moxifloxacin and pyrazinamide was recommended for all HIV-positive contacts once active disease was ruled out. Among exposed residents, 40 (48%) either died of non–TB-related causes or were lost to follow-up before completing TB evaluation, and 12 (14%) either refused treatment or were not started on treatment because of physician decision. Of the remainder, 24 initiated moxifloxacin and pyrazinamide treatment; 14 (58%) completed treatment, and 10 (42%) refused or were lost-to-follow up after a median 3 (range 1–5) months of treatment. The 2 contacts whose TST results were converted were placed on alternative regimens.

To determine whether TB symptoms subsequently developed in any contact in either outbreak, we compared them to cases identified in the NYC TB registry. As of March 2014, after a maximum of 8.5 years of follow-up at site A and 9 years at site B, 1 contact, a resident at site B who completed 1 month of moxifloxacin and pyrazinamide treatment in 2006 had TB disease caused by a different drug-susceptible strain develop during 2009.

## Conclusions

Globally, an estimated 480,000 persons were infected with MDR TB in 2013 (World Health Organization Global Tuberculosis Report 2013, http://apps.who.int/iris/bitstream/10665/91355/1/9789241564656_eng.pdf). Although the current priority is accurate diagnosis and treatment of persons with active disease, preventing MDR TB in infected contacts is also crucial. Programs with capacity for contact investigation are faced with the question of what prophylaxis to recommend for persons who have contact with persons who are positive for MDR TB because data are scarce on efficacy and safety of treatment options for these persons.

Currently recommended treatment regimens (Table 1) are not without risk. Studies demonstrate serious adverse effects associated with the use of pyrazinamide in combination with either ethambutol (*10*) or a fluoroquinolone, including ofloxacin (*11*,*12*) and levofloxacin (*13*,*14*). However, a recent study of contacts treated with moxifloxacin- or levofloxacin-based prophylaxis found no serious adverse events and fewer cases of disease among those treated than those untreated (*15*).

Of the 50 contacts initiating moxifloxacin-based prophylaxis in the 2 outbreaks, 30 (60%) completed treatment. Therapy was generally well-tolerated; 3 contacts discontinued treatment because of gastrointestinal symptoms, (nausea, vomiting, diarrhea). None of the contacts manifested TB symptoms regardless of treatment status. Those contacts at greatest risk for development of disease may have done so during the outbreak investigation.

Because of the lack of safety information for moxifloxacin-based prophylaxis, we reported these findings despite key limitations. These outbreak investigations were conducted as part of routine TB control activities; thus, treatment regimens were not standardized across the study populations, and contacts were not actively followed. However, these outbreaks demonstrate known exposure to infectious MDR TB case-patients with strong evidence of transmission to a high-risk HIV-positive population. Because of robust disease reporting and broad surveillance coverage, we are able to report on outcomes in NYC within <9 years of follow-up time. This study demonstrates that moxifloxacin-based regimens can be used to treat HIV-positive persons exposed to MDR TB with few serious adverse effects, although we were unable to assess efficacy of the regimen. More research is needed to evaluate the effectiveness of prophylaxis for MDR TB.

## References

[1] American Thoracic Society. Targeted tuberculin testing and treatment of latent tuberculosis infection. Am J Respir Crit Care Med. 2000;161:S221–47 and. 10.1164/ajrccm.161.supplement_3.ats600 10764341

[2] Curry International Tuberculosis Center and California Department of Public Health. Drug-resistant tuberculosis: a survival guide for clinicians. 2nd ed. San Francisco; 2011 [cited 2014 Dec 19]. http://www.currytbcenter.ucsf.edu/drtb/

[3] European Centre for Disease Prevention and Control. Management of contacts of MDR TB and XDR TB patients. Stockholm: The Centre; 2012 [cited 2014 Dec 19]. http://www.ecdc.europa.eu/en/publications/Publications/201203-Guidance-MDR-TB-contacts.pdf

[4] Centers for Disease Control and Prevention. Management of persons exposed to multidrug-resistant tuberculosis. MMWR Recomm Rep. 1992;41(RR-11):61–71 . 1640921

[5] New York City Department of Health and Mental Hygiene Bureau of Tuberculosis Control. Tuberculosis: clinical policies and protocols. 4th ed. New York: The Department; 2008 [cited 2014 Dec 19]. http://www.nyc.gov/html/doh/downloads/pdf/tb/tb-protocol.pdf

[6] World Health Organization. Guidelines on the management of latent tuberculosis infection. Geneva: The Organization; 2015 [cited 2014 Dec 19]. http://apps.who.int/iris/bitstream/10665/136471/1/9789241548908_eng.pdf?ua=1 25973515

[7] Sharma SK, Mohan A, Kadhiravan T. HIV–TB co-infection: epidemiology, diagnosis & management. Indian J Med Res. 2005;121:550–67 . 15817963

[8] Markowitz N, Hansen NI, Hopewell PC, Glassroth J, Kvale PA, Mangura BT, Incidence of tuberculosis in the United States among HIV-infected persons. The Pulmonary Complications of HIV Infection Study Group. Ann Intern Med. 1997;126:123–32 and. 10.7326/0003-4819-126-2-199701150-00005 9005746

[9] Schluger NW, Burzynski J. Tuberculosis and HIV infection: epidemiology, immunology, and treatment. HIV Clin Trials. 2001;2:356–65 and. 10.1310/TUNH-UAKU-N0E4-1PXF 11590540

[R10] World Health Organization. Global tuberculosis report 2013. Geneva: The Organization; 2014 [cited 2014 Dec 19]. http://apps.who.int/iris/bitstream/10665/91355/1/9789241564656_eng.pdf

[10] Younossian AB, Rochat T, Ketterer JP, Wacker J, Janssens JP. High hepatotoxicity of pyrazinamide and ethambutol for treatment of latent tuberculosis. Eur Respir J. 2005;26:462–4 and. 10.1183/09031936.05.00006205 16135729

[11] Ridzon R, Meador J, Maxwell R, Higgins K, Weismuller P, Onorato IM. Asymptomatic hepatitis in persons who received alternative preventive therapy with pyrazinamide and ofloxacin. Clin Infect Dis. 1997;24:1264–5 and. 10.1093/clinids/24.6.1264 9195099

[12] Horn DL, Hewlett D Jr, Alfalla C, Peterson S, Opal SM. Limited tolerance of ofloxacin and pyrazinamide prophylaxis against tuberculosis. N Engl J Med. 1994;330:1241 and. 10.1056/NEJM199404283301718 8139647

[13] Papastavros T, Dolovich LR, Holbrook A, Whitehead L, Loeb M. Adverse events associated with pyrazinamide and levofloxacin in the treatment of latent multidrug-resistant tuberculosis. CMAJ. 2002;167:131–6 . 12160118 PMC117089

[14] Adler-Shohet FC, Low J, Carson M, Girma H, Singh J. Management of latent tuberculosis infection in child contacts of multidrug-resistant tuberculosis. Pediatr Infect Dis J. 2014;33:664–6 and. 10.1097/INF.0000000000000260 24445837

[15] Bamrah S, Dorina F, Setik L, Song R, Kawamura LM. A. Heetderks A, et al. Treatment for LTBI in contacts of MDR-TB patients, Federated States of Micronesia, 2009–2012. Int J Tuberc Lung Dis. 2014;18:912–8. 10.5588/ijtld.13.0028 PMC4730114 25199004

